# Beyond The Basics: Unveiling Superior Vena Cava Compression in Hodgkin’s Lymphoma

**DOI:** 10.7759/cureus.74434

**Published:** 2024-11-25

**Authors:** Shivendra Tangutoori, Dedeepya Gullapalli, Sai S Kommineni, Avinash Vangara, Kamlesh Sajnani, Subramanya Shyam Ganti

**Affiliations:** 1 Internal Medicine, Appalachian Regional Healthcare, Harlan, USA; 2 Hematology and Oncology, Appalachian Regional Healthcare, Harlan, USA; 3 Internal Medicine/Pulmonary Critical Care, Appalachian Regional Healthcare, Harlan, USA

**Keywords:** abvd chemotherapy, anterior mediastinal mass, hematology-oncology, hodgkin’s lymphoma, hodgkin’s lymphoma presenting with eosinophilia, horner’s syndrome, right sided pleural effusion, superior vena cava (svc) syndrome, unique case

## Abstract

Hodgkin’s lymphoma (HL) is a malignancy of the lymphocytes in the lymph nodes and presents with non-specific systemic symptoms like fever, night sweats, and weight loss. While HL often involves the mediastinum, it rarely causes superior vena cava (SVC) syndrome, and eosinophilia is noted in approximately 15% of cases. Here, we report a unique presentation of HL in a 52-year-old male with a history of chronic pruritus, chronic kidney disease, and inactive hepatitis B. The patient presented with progressive cough and dyspnea over an eight-month period and developed right-sided ptosis, neck swelling, and engorged veins in the chest, suggesting Horner's syndrome and SVC syndrome. Imaging revealed an enlarged mediastinal mass and a pleural effusion, with subsequent biopsy confirming classical HL. Notably, the patient exhibited severe eosinophilia and a pruritic rash, which are uncommon features seen with HL. Following a multidisciplinary discussion, he was diagnosed with stage IV B x (bulky) HL and was started on a chemotherapy regimen with brentuximab vedotin plus AVD, which led to significant symptom resolution and reduction in tumor size. This case highlights the importance of considering HL in patients presenting with chronic pruritus, eosinophilia, and mediastinal masses. It emphasizes the need for comprehensive diagnostic evaluation in challenging cases and underscores the effectiveness of modern chemotherapy regimens in managing advanced stages of HL.

## Introduction

Hodgkin’s lymphoma (HL) is a malignant lymphoma, which is seen in the lymph nodes and its prognosis has improved in the last 20 years [1]. HL involves the mediastinum more commonly than other lymphomas but rarely causes superior vena cava (SVC) syndrome (SVCS) [2]. Pruritus arises as a primary dermatologic condition or a manifestation of a broad array of systemic diseases [3]. Pruritus with severe eosinophilia requires a comprehensive evaluation for an accurate diagnosis. HL is associated with eosinophilia in 15% of cases [4]. This case report discusses the rare occurrence of HL presenting with eosinophilia and a pruritic rash, SVCS, and Horner’s syndrome.

## Case presentation

A 52-year-old man with a past medical history of chronic kidney disease, chronic pruritus, and inactive chronic hepatitis B infection presented with progressive cough and shortness of breath for eight months. The cough was productive with a clear sputum associated with occasional wheezing. He had no postnasal drip, orthopnea, paroxysmal nocturnal dyspnea, pedal edema, hemoptysis, fever, or chills. He had episodes of passing out following a severe coughing spell. The patient had been on prednisone 10 mg daily for five years for the chronic pruritus which was earlier diagnosed as eczema. He had never smoked but had a remote history of intravenous drug use; he had been in remission for eight years. On general examination, the patient appeared to be in mild discomfort. He was afebrile, with a blood pressure of 142/90 mmHg, a pulse of 68 beats per minute, a respiratory rate of 22 breaths per minute, and an oxygen saturation of 98% while breathing ambient air. No pallor, jugular venous distension, lymphadenopathy, pedal edema, or clubbing was noted. Laboratory studies showed leukocytes of 18,620 cells/µL (4,230 to 9,070 cells/µL) with eosinophils of 41.7% (0.8% to 7.0%) with a count of 7,760 cells/µL (30-350 cells/µL); otherwise, hemoglobin, platelet count, serum electrolyte, and liver function tests were normal (Table 1).

**Table 1:** Complete blood count of the patient on initial presentation WBC: white blood corpuscle; RBC: red blood corpuscle; uL: microliter.

Cell type	Result	Reference range
WBCs	10.86	4.23-9.07 (x10^3^/uL)
RBCs	5.24	4.63-6.08 (x10^6^/uL)
Hemoglobin	16.4	13.7-17.5 g/dL
Platelet count	177	163-337 (x10^3^/uL)
Neutrophils	46.5	34.0-67.9%
Lymphocytes	5.2	21.8-53.1%
Monocytes	5.6	5.3-12.2%
Eosinophils	41.7	0.8-7.0%
Basophils	0.7	0.2-7.0%

**Figure 1:**
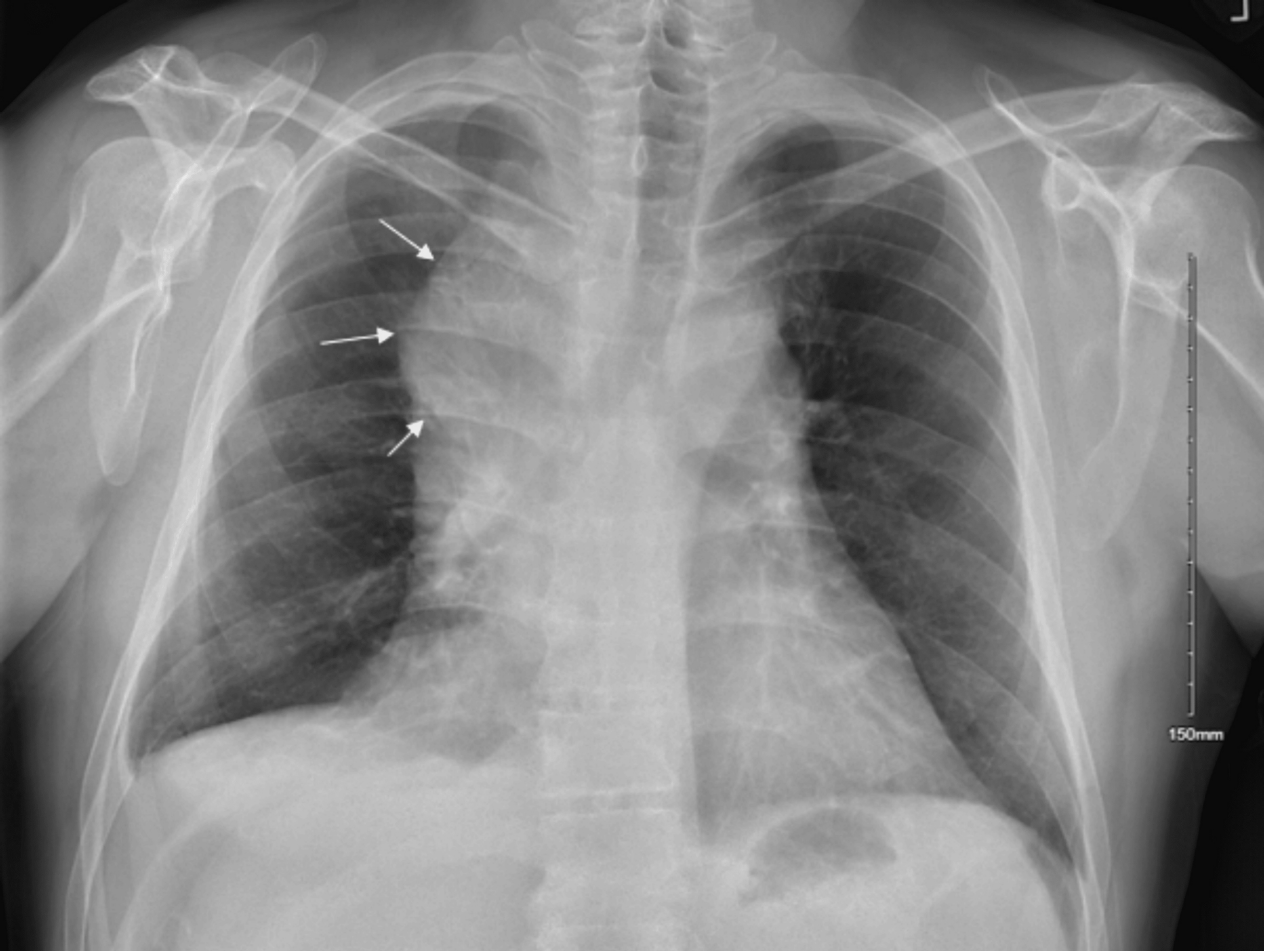
Chest radiograph of the patient showing right-sided suprahilar mass-like lesion.

A peripheral smear obtained showed marked eosinophilia with no circulating blasts. Further testing for peripheral eosinophilia including Strongyloides IgG antibody, Toxocara antibody, and serum tryptase levels were all negative. Chest X-ray (Figure 1) showed a right-sided mass like suprahilar parenchymal density, recommended to follow up with computerized tomography (CT) of the chest.

**Figure 2:**
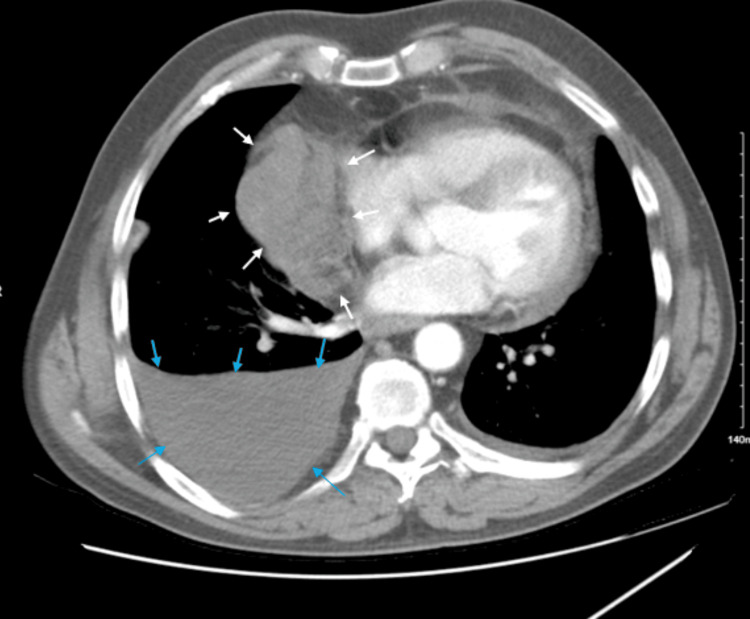
CT scan of the chest showing ‘soft tissue mass’ shown by the white arrows and the ‘pleural effusion’ noted by the blue arrows. CT: computed tomography.

CT chest (Figure 2) showed a 10.5 x 8.6 x 18.7 cm soft-tissue density mass lesion involving the right aspect of the mediastinum which narrows and surrounds the adjacent vasculature, consistent with neoplasm. There were borderline enlarged perivascular lymph nodes present measuring up to 14 mm. A moderate amount of right-sided pleural effusion was noted.

**Figure 3:**
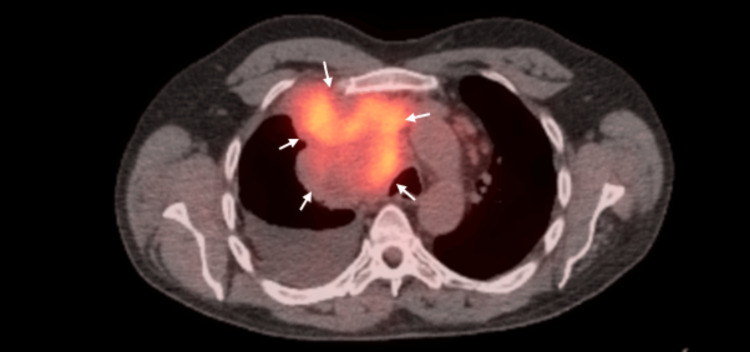
PET/CT showing a hypermetabolic mediastinal mass as indicated by the arrows. PET/CT: positron emission tomography/computed tomography.

The patient had thoracentesis done with pleural fluid analysis showing a lactate dehydrogenase level of 142 IU/L, total protein of 4.6 g/dL, and pleural cholesterol level of 117 mg/dL, supporting an exudative type of pleural effusion as per Light’s criteria and Heffner’s criteria. Pleural fluid cytology revealed reactive mesothelial cells with eosinophil prominence and negative for malignant cells. PET/CT imaging (Figure 3) revealed a large hypermetabolic mass in the medial aspect of the right hemithorax with a maximal SUV of 5.6 and a hypermetabolic subpleural pulmonary nodule in the right lung base with a maximal SUV of 3.2. Multiple hypermetabolic lymph nodes were also seen on the anterior-posterior window.

The patient had a bronchoscopy and endobronchial ultrasound (EBUS) with fine-needle aspiration, during which enlarged lymph nodes were identified at station 7, subcarinal lymph node at station 11R, and right lung mass was identified at station 4R extending into 10R. The subcarinal lymph node (station 7) was sampled under ultrasound guidance with a total of five needle aspirations along with the endobronchial biopsy of the mass being obtained; one transbronchial needle aspiration was done at station 11R, and the pathology did not reveal any malignant cells. Four weeks after the PET/CT imaging, he underwent rigid bronchoscopy, EBUS, radial EBUS, and transbronchial biopsy performed by the interventional pulmonology at the higher center. A repeat biopsy of lymph nodes was done and the 11R station lymph node showed CD30-positive cells. The right upper lobe mass and the station 7 lymph node showed rare, atypical cells, numerous eosinophils, and scattered lymphocytes. A discussion with the tumor board committee was held and the decision was made to pursue a bone marrow biopsy with an aspirate. Analysis of the bone marrow aspirate showed a large number of eosinophils with no blasts or abnormal lymphoid population.

The patient presented to the pulmonology clinic three months after his bronchoscopy with worsening dyspnea, associated with ptosis on the right side with swelling in the neck and engorged veins on the chest. The patient developed right axillary and supraclavicular lymphadenopathy along with worsening skin rash/eczema. He had increased oxygen requirements and was placed on a 2 L nasal cannula. Bilateral diffuse diminished breath sounds were noted. Neurologic and musculoskeletal examinations were normal.

**Figure 4:**
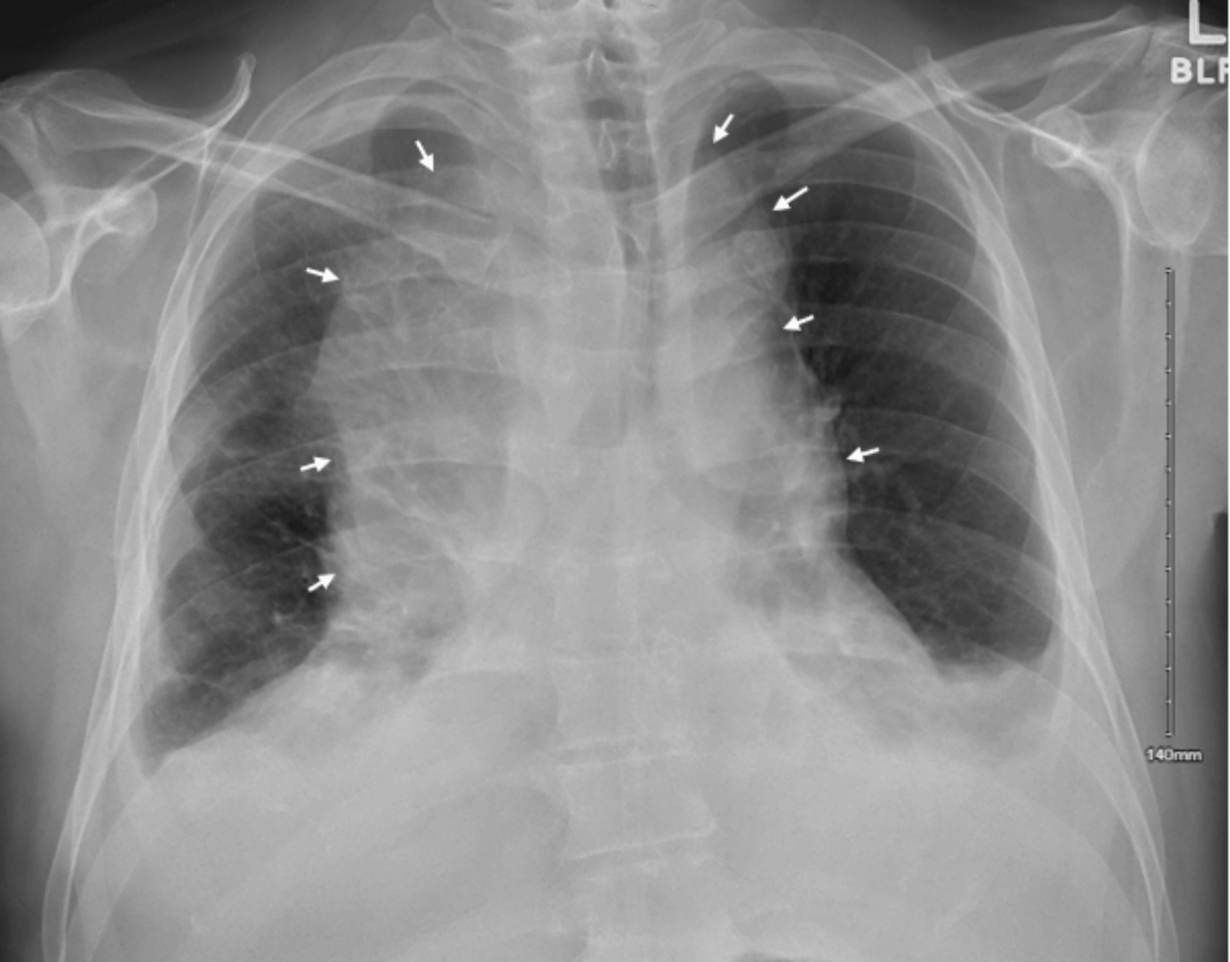
Chest radiograph showing worsening right-sided suprahilar parenchymal density/mass.

A chest radiograph (Figure 4) was obtained and compared with previous films, which showed an increasing right suprahilar parenchymal density.

CT-guided biopsy of the mediastinal mass was done later and it revealed classical HL (cHL) with rare binucleated cells that expressed molecular markers CD30, dim PAX-5, and MUM-1. Immunohistochemistry staining was negative for CD20, CD45, CD15, and ALK1. Epstein-Barr virus in situ hybridization stain was negative. On CD138 and IgG4 stains, there was no significant increase in plasma expressing IgG4.

**Figure 5:**
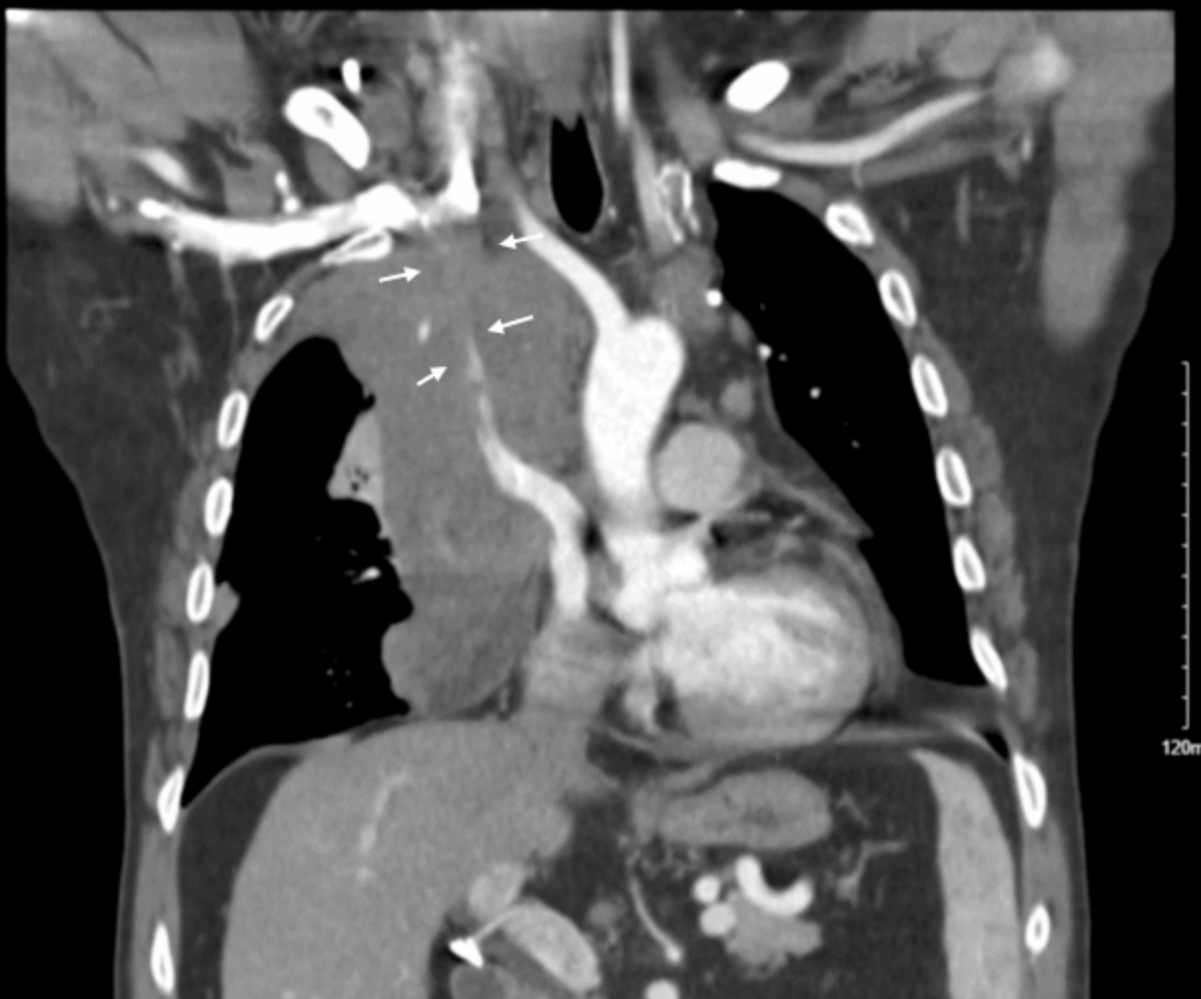
CT scan showing tumor compressing SVC causing compressive symptoms. CT: computed tomography; SVC: superior vena cava.

After a diagnosis of HL, the patient developed compressive symptoms (Figure 5) of right-sided ptosis, miosis, and anhidrosis suggesting Horner’s syndrome along with systemic symptoms of weight loss of 12 lbs and loss of appetite. He also developed vein engorgement with collaterals formed across the chest and neck regions suggesting SVCS.

**Figure 6:**
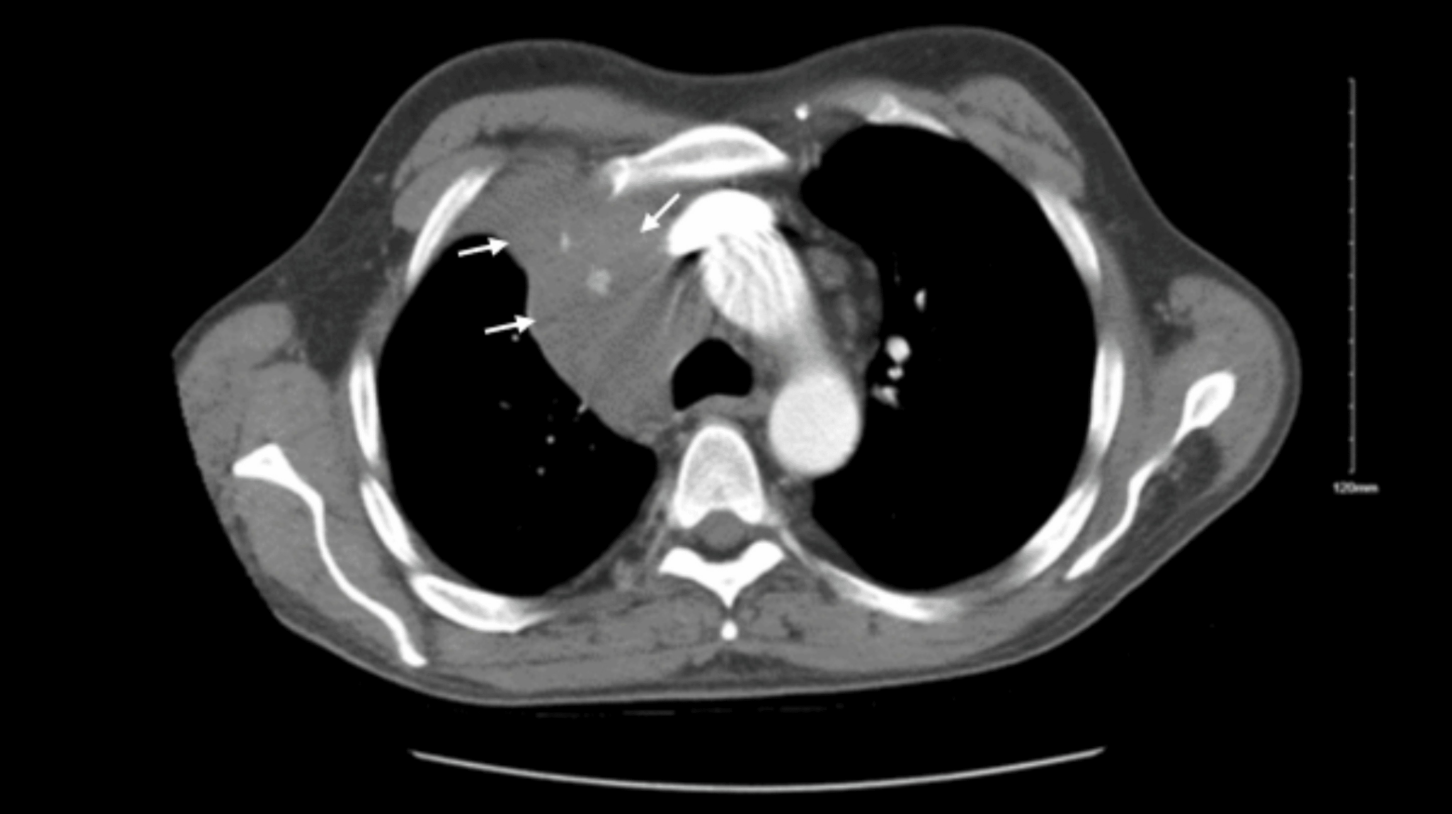
CT scan of the chest, six months post-treatment, showing a reduction in the size of the lymphoma. CT: computed tomography.

He was immediately referred to oncology services and was classified as stage 4Bx HL. A chemotherapy regimen with brentuximab vedotin plus AVD (doxorubicin, vinblastine, and dacarbazine) was initiated, which led to significant improvement and immediate resolution of compressive symptoms and repeat CT scan six months post-treatment showed a significant reduction in the size of lymphoma (Figure 6).

## Discussion

HL is a rare monoclonal lymphoid neoplasm that has a bimodal distribution of incidence affecting young adults as well as patients older than 55 years [1]. A few of the risk factors include family history, viral infection, autoimmune diseases, and immunosuppressed states [1]. Familial involvement suggests a genetic cause whereas an abnormal immune response to infection can also play a role in the pathogenesis of HL, especially EBV [5]. Patients with HIV have a higher risk of HL when compared to the general population [6]. Based on the histology and immunohistochemical analysis from the lymph node biopsy, HL is classified into two distinct categories: cHL and nodular lymphocyte-predominant (LP) HL. cHL is further divided into four subgroups: nodular sclerosis, lymphocyte-rich, lymphocyte-depleted, and mixed cellularity [7]. cHL is B cell lymphoma lacking the B cell phenotype due to the hypermutation and c-ass switched immunoglobulin genes causing non-functional immunoglobulin lacking the B cell receptor expression. In a healthy B cell, such mutations should lead to apoptosis but in HL, the cells are spared from apoptosis [8]. Most people present with supradiaphragmatic lymphadenopathy; inguinal areas are less frequently involved. One-third of the patients present with systemic symptoms like fever, night sweats, weight loss, and chronic pruritus. Extranodal organ involvement can include the spleen, lung, liver, and bone marrow either through hematogenous spread or by direct invasion from the nodes [9]. HL presenting as SVCS, that is, compression or obstruction of SVC (SVCO) causing distended neck veins, cough, orthopnea, and dyspnea, occurs in less than 2% of affected patients [10]. SVCS can develop before, during, or after the therapy for HL. Because of the hypercoagulability, HL can contribute to late-onset SVCS. Diagnosis of HL presenting as SVC syndrome is complex since SVCO symptoms such as cough, dyspnea, and orthopnea overlap with a wide range of respiratory and cardiovascular conditions; secondly, HL is characterized by large mediastinal masses that can mimic other malignancies or infectious diseases in the presence of non-specific symptoms like pruritus and eosinophilia, as in our patient. Because of the limited data and rare prevalence, the prognosis of HL with SVCS presentation remains difficult [10]. The incidence of eosinophilia in HL is approximately 15% [4]. Both peripheral and tissue eosinophilia have been noted in HL. 

Eosinophils have been noted to have an important role in the pathobiology of HL. ​The mechanism of eosinophilia remains unknown, though various mediators like interleukin 5 (IL-5) and granulocyte-macrophage colony-stimulating factor (GM-CSF) have been implicated​. Eosinophilia may be either mild, moderate, or severe based on the number of eosinophils. Eosinophilia could indicate various other pathologies including both infectious and non-infectious diseases. Infectious etiology includes fungal infections like coccidioidomycosis, histoplasmosis, and aspergillosis, bacterial or mycobacterial infections, viral infections like human immunodeficiency virus, human T-lymphotropic virus type 1, or Epstein-Barr virus, and parasitic infections like Ascaris, Strongyloides, and Toxocara. Non-infectious etiology includes asthma or allergic diseases, rheumatologic or connective tissue disease, adrenal insufficiency, or hematologic or neoplastic disease, which includes lymphoma, eosinophilic leukemia, or mastocytosis. Patients with malignant eosinophilic pleural effusions have a better prognosis than those with non-eosinophilic pleural effusion malignancies; higher eosinophilic count is shown to improve overall survival rates among malignant eosinophilic pleural effusions [11].

In the management of SVCS, the goals are to relieve symptoms and to attempt a cure for the primary malignant process. Elevation of the bed is usually recommended with supplemental oxygen. Corticosteroids and diuretics are used to relieve laryngeal or cerebral edema, although documentation of their efficacy is questionable. Radiotherapy has always been advocated as a standard treatment for most patients with SVCS but chemotherapy is preferable to radiation for patients with chemosensitive tumors [12], once the tissue diagnosis has been established as in our patient. When SVCS is due to a thrombus around a central venous catheter, patients may be treated with thrombolytics or anticoagulants. Endovascular stenting is used for severe or refractory cases to relieve obstruction. Several scoring systems have been introduced to assess the acuity of the obstruction and symptoms. Scoring systems such as the Kishi Scoring System, Yale Grading System, and Stanford Classification for SVCS have been used to grade the clinical severity for guiding further treatment management, especially endovascular stent placement [13]. For example, the Kishi scoring system uses the patient’s neurologic symptoms, laryngopharyngeal or thoracic symptoms, nasal and facial signs or symptoms, and the presence of venous dilatation and scores them from 0 to 10, with a score of 4 or greater requiring stent placement. Our patient has a score of 1 based on this scoring system which can be managed by the above-mentioned modalities and does not require a stent placement.

The diagnosis of HL made only with a biopsy of the lymph node either by fine-needle aspiration or core needle can sometimes be inconclusive due to the varying architecture of the lymph node. HL is unique as the tumor cells constitute the minority of the cell population in the lymph node and inadequate biopsy sampling can fail to establish the definitive diagnosis of the malignancy [14]. An excisional lymph node biopsy is preferred for a wholesome analysis and to identify Reed-Stenberg cells or LP cells to definitely diagnose HL. Staging of HL is done by the modified Ann-Arbor classification. Early-stage favorable cHLs (stage I to IIa) with favorable prognostic features are treated with two cycles of doxorubicin, bleomycin, vinblastine, and dacarbazine (ABVD) followed by restricted involved-field radiation therapy. Advanced-stage cHL is treated with a combination of four cycles of chemotherapy and consecutive radiation. Advanced-stage cHL is treated with six cycles of ABVD and consecutive PET-adopted radiation therapy of residual lesions [15]. A newer regimen eliminating bleomycin and substituting brentuximab vedotin (an anti-CD30 monoclonal antibody linked to the anti-tubulin agent monomethyl auristatin E) is associated with less pulmonary toxicity. It has slightly improved outcomes for patients with advanced disease. ​Radiation therapy to stages III and IV is given after chemotherapy, if there are any large tumor areas or in case of resistant or refractory HL. As our patient responded well to the chemotherapy, radiation therapy was not considered.

## Conclusions

The unusual presentation of HL with chronic pruritus, eosinophilia, SVCS, and Horner’s syndrome is highlighted in this case report, emphasizing the unique but significant diversity of its clinical manifestations and why it is crucial to rule out HL in patients exhibiting the above clinical features.

Despite the complicated presentation and diagnostic challenge, focused treatment with advanced modern treatment regimens can result in significant clinical advantages, as seen by eliminating compressive symptoms and overall improvement following chemotherapy. It also highlights how important comprehensive diagnostic evaluation and individualized treatment plans are to the management of complicated HL cases.
